# Measuring social interdependence in collaborative learning: instrument development and validation

**DOI:** 10.1186/s12909-020-02088-3

**Published:** 2020-06-01

**Authors:** Ikuo Shimizu, Makoto Kikukawa, Tsuyoshi Tada, Teiji Kimura, Robbert Duvivier, Cees van der Vleuten

**Affiliations:** 1 Center for Medical Education and Clinical Training, Shinshu University, 3-1-1 Asahi, Matsumoto, 3908621 Japan; 2 Department of Medical Education, Kyushu University, 3-1-1 Maidashi Higashi-ku, Fukuoka, 8128582 Japan; 3 Department of Fundamental Physical Therapy, Shinshu University, 3-1-1 Asahi, Matsumoto, 3908621 Japan; 4 Center for Educational Development and Research in Health Sciences (CEDAR), University Medical Center Groningen, Groningen, The Netherlands; 5 Parnassia Psychiatric Institute, The Hague, The Netherlands; 6 Department of Educational Development and Research, Faculty of Health, Medicine and Life Sciences, Maastricht University, Universiteitssingel 60, 6229 ER Maastricht, The Netherlands

**Keywords:** Social interdependence, Collaborative learning, Instrument, Validation

## Abstract

**Background:**

In health professions education, several collaborative learning approaches have been used. As collaborative learning has a theoretical background of social interdependence theory, a theory informed and valid instrument to measure social interdependence is required to evaluate and compare several learning approaches. The aim of this study was to develop an instrument (the SOcial interdependence in Collaborative learning Scale; SOCS) to measure students’ perceived social interdependence in collaborative learning and validate it.

**Methods:**

We conducted a modified Delphi procedure among stakeholders to develop the content validity of the instrument. To establish construct validity, we performed a confirmatory factor analysis, and we estimated reliability.

**Results:**

Two rounds of Delphi were conducted to develop the instrument. Confirmatory factor analysis yielded a three-factor model with 15 items, which provided an excellent fit with CMIN/df = 1.838, GFI = 0.924, CFI = 0.951, RMSEA = 0.061, and PCLOSE = 0.121. Alpha-coefficients for all factors indicated high internal consistency of all the factors.

**Conclusion:**

This study describes the development and construct validation of the SOCS for measuring social interdependence in collaborative learning. This instrument will provide teachers and schools with feedback about their classroom environment.

## Background

### Collaborative learning in health professions education

The development of small group learning in health professions education has occurred because of the evidence indicating that students in small groups exceed their counterparts in several key areas [1]. These include knowledge achievement, thinking skills, and social skills. Especially for health professions education, the increasing complexity of health problems demands the expertise of, contributions from, and participation by, each of the professionals on the team [2]. Therefore, physicians must be competent collaborators with team members to practice medicine effectively. Because students typically have minimal contact with each other in the process of their education, medical schools have tried to implement a variety of group learning experiences into their preclinical as well as clinical curriculums.

Currently, one of the common forms of group learning is collaborative learning [3]. Collaborative learning is characterised by not only students working together in groups, but also the group working together with the teacher to develop knowledge, thus shifting the nature of authority in the classroom [4]. There are also some commonalities across these group work approaches between collaborative learning and cooperative learning, an older form of group learning. In a theoretical synthesis of cooperative and collaborative learning approaches, several studies have tried to explore interactions taking place between students in groups. However, collaborative learning subsumes cooperative learning and extends it by emphasizing substantial dialogue and co-construction of ideas [5], and typical examples that involve these chracteristics are problem-based learning (PBL) and team-based learning (TBL) [6]. In addition, collaborative learning is also used for teamwork of multiple professionals. To prepare students for effective interprofessional clinical work (i.e. interprofessional education), learning approaches in small groups enabling professions to interact with each other is warranted [7].

Five attributes are common to collaborative group learning approaches: a common task suitable for group work; small-group interaction focused on the learning activity; cooperative, mutually helpful behaviour among students; individual accountability and responsibility; and interdependence in working together [5]. It has been pointed out that positive social interdependence is a fundamental attribute in collaborative group learning approaches, because the outcomes of beneficial interdependence are associated with multiple and far-reaching effects on students’ learning experiences [8]. For example, research has shown that group learning does not yield higher achievement [9] and productivity [10] without the impact of positive social interdependence.

### Social interdependence

Social interdependence theory (SIT) has become one of the most widespread applications of educational psychology [8]. Social interdependence exists when the outcomes of individuals are affected by their own and others’ actions [11]. There are two types of social interdependence: positive (i.e. the actions to promote the achievement of joint goals) and negative (i.e. the actions to obstruct the achievement of each other’s goals). The psychological processes based on positive interdependence include substitutability (the degree to which actions of one person substitute for the actions of others), inducibility (i.e. openness to being influenced and to influencing others), and positive cathexis (i.e. investment of positive psychological energy in objects outside of oneself) [8]. These processes demonstrate how self-interest is expanded to joint interest, and how new goals and motives are created in cooperative and competitive situations. The transformation from self-interest to mutual interest is one of the most important aspects of social interdependence.

The process to structure positive and negative interdependence is divided into three categories: outcome, means, and boundary [11]. Outcome interdependence stands for orientation towards goal and reward. Goals can be real or fantasized and structuring positive outcome interdependence often results in increased achievement and productivity [12]. For example, if group members in PBL grasp what they would achieve through the session, it would be more productive.

Means interdependence includes resource, role, and task interdependence. Resources (e.g. room, blackboard, writing materials, computers and other information devices) are used among group members, some of which are prepared as joint property. Roles are assigned to group participants such as reader, recorder, summarizer, and encourager. As per task interdependence, if the assigned tasks can be divided based on the mutual agreement of group members, each group member is responsible for doing one aspect of the assignment, which will lead the learning group more productive.

Boundary interdependence is based on abrupt discontinuities among individuals, and thus includes outside enemy (negative interdependence among groups), identity (categorizing group members as an entity), and environmental interdependence (such as a working area) [8] because boundaries between individuals and groups can define who is interdependent with whom. It may exist due to abrupt discontinuities among individuals that segregate individuals into separate groups, while the discontinuity depends on environmental factors, similarity, proximity, expectations of being grouped together, and differentiation from other groups.

Positive interdependent cooperation does not only tend to result in more frequent use of higher-level reasoning and more intrinsic motivation, but also promotes more positive interpersonal relationships and greater social support [11]. Also, there is evidence that cooperation in learning procedures promotes more positive attitudes toward learning subjects than competitive or individualistic learning [11]. These outcomes resulting from collaborative efforts tend to be reciprocally related [11]. In other fields than pedagogy, research shows that positive interdependence improves working outcomes in Non-government organizations [13] and job management [14, 15]. Above all, clinical workplace is one of the settings where social interdependence has been rapidly warranted due to its recent drastic change within the decade [16]. Different from other workplaces, it is characteristic that health professions have to construct more numerous and complex relations between intraprofessional and interprofessional care providers, trainees and trainers, health professions and patients. Therefore, educators need to develop social interdependent attitude among learners and then assess it for competence judgement [17].

The interest of interdependence in collaborative learning causes another concern about how to evaluate interdependence. While there is a need to clarify the interactions within collaborative learning approaches to design and implement more effectively, few studies have been conducted regarding the quality of interaction, including interdependence for PBL and other group learning approaches [18], and there has been no validated instrument that can measure three categories of social interdependence (i.e. outcome, means, and boundary) in collaborative learning for health professions education. A new instrument for assessing different categories of interdependence within collaborative learning would enable us to analyse and enhance our collaborative learning approaches, and improve students’ interaction better and achieve more educational outcomes.

Therefore, the purposes of this study were to develop a new instrument for measuring students’ perceived social interdependence in collaborative learning to assess by establishing consensus among stakeholders, and validate the instrument by demonstrating how those who participated in collaborative learning class responded to the instrument.

## Methods

### Instrument development phase

In order to develop an instrument with good content validity for evaluating social interdependence, we conducted a modified Delphi procedure. It is an interactive process designed to establish consensus on specific criteria or items by gathering opinions from professionals in the field [19] and has evidence for the content validity of research products [20]. This process aims to build consensus among experts in a systematic manner and consists of multiple consultation rounds in which the experts indicate their agreement with statements or concepts [21]. The inclusion of different stakeholders in a Delphi procedure promotes acceptance of feedback and effective implementation of the product [22]. Therefore, we decided to include three groups of stakeholders: faculty, educational experts and students.

### Preparation for the first Delphi round

We started by procuring items for a questionnaire with regard to social interdependence from a literature search. In September 2017, the first author (IS) independently searched Google Scholar for English-language papers using Boolean key words. Combinations of search terms were used, relating to social interdependence (interdependence, collaboration, cooperation) and instrument (survey, questionnaire). We intended that the items of the initial list should relate to questionnaires which were previously used for various fields, especially in view of three categories (outcome, means, boundary). Through the literature search, five articles regarding social interdependence across a number of educational contexts were identified [18, 23–26]. The two authors (IS and MK) discussed and agreed that 86 prospective items were included. We decided that these items should relate to our purpose as these were easier for students to answer. Then the items that were considered to be similar were edited from the prospective items to an initial list of 37 items. While the list was originally developed in English, a Japanese version was also developed by back translation for some panelists whose academic language is Japanese.

### Recruitment of panelists

We selected panelists to ensure representation of three groups of stakeholders: 10 medical students, 10 education experts, and 10 medical educators with any experience of collaborative learning in 8 countries (Australia, Czech Republic, Japan, Malaysia, the Netherlands, Singapore, Thailand, the United States), considering the diversity of cultures [27]. We took a recommendation into consideration to decide the number of total panelist [28]. During selection, we tried to ask heterogeneous panels, characterized by members in various geographic contexts with essentially different perspectives on education are likely to produce a higher proportion of high quality and highly acceptable solutions than homogeneous groups [27]. The education experts were purposefully selected based on their knowledge on health professions education: those who had master or higher degrees of health professions education were selected. The medical educators all had experience on collaborative learning such as PBL and TBL in a health professions education curriculum. For medical students, all were at their clinical years and had experience on participating in a collaborative learning class previously. Participation was voluntary, participants received no reward and the data were anonymized.

### Distribution of survey

We sent the online list at surveymonkey.com to the panelists and asked them to rate each item on a five-point Likert scale (1 = unimportant, 2 = of little importance, 3 = neutral, 4 = relevant, 5 = very relevant), and calculated means and standard deviations. We also asked panelists to change redundant or unnecessary phrases, and suggest additional items to edit the list in accordance with their comments.

### Criteria for inclusion of items in the instrument

While there are no definite rules to decide when consensus is achieved in Delphi rounds, we used the two following indicators in previous articles to determine whether consensus was achieved [19, 29]:

1. The mean value of 3.0 (the midpoint of the five-point scale) was applied as an indicator of group consensus.
2. The standard deviation (SD) was applied to evaluate the dispersion of responses for each criterion and provide further evidence of consensus (i.e. The smaller the SDs, the greater the consensus). Those criteria with an SD of less than 1.25 was deemed to indicate consensus and considered to be a positive criterion for inclusion in the instrument.

We also referred to the panelists’ ratings and tried to keep the questionnaire manageable. If multiple panelists suggested similar additional items, an additional Delphi round would be conducted.

As a result, we selected 16 items for the rounds.

### Validation phase

After completing the questionnaire, we conducted the validation study with fourth year health professions students undertaking a collaborative learning class in Shinshu University, in the six-year (medicine) or four-year (nursing, physical therapy, occupational therapy, medical technology) curriculum. In this class, which was held in the autumn semester of 2017, students were randomly divided into groups of 7–8 from the four disciplines, and were asked to work out a long clinical case scenario of a patient with amyotrophic lateral sclerosis, including the diagnosis, progression, and end-stage. They were expected to apply their knowledge of each discipline. They sat at the same table for analysis and problem solving of the case and submitted the results of their discussions as assignments. Finally, they made a presentation about the assignments of the group and discussed with other groups. In other words, they experienced all types of social interdependence. Tutors, all of whom were involved in the development and coordination of the class, ensured as that students identified issues within and outside the group discussions that impact on learning.

In December 2017, we asked all the students (*n* = 264) engaged in the class to complete the questionnaire at the end of the class. Students received a written informed consent form, and those who understood the purpose of the research and agreed to participate received the questionnaire. Questionnaires were administered anonymously. Participants were informed that the survey was not mandatory and was not related to their grading. Students were given about 10 min to answer the questionnaire.

### Analysis

We determined the construct validity of the instrument. First, we calculated skewness (tilt in the distribution) and kurtosis (peakedness of the distribution) to check the normality of the distribution. Since the skew and kurtosis values of all the data we used were smaller than 1.5, they were normally distributed. Thus it was appropriate to use a maximum likelihood estimation to conduct the factor analyses. We performed exploratory factor analysis (EFA) for uncovering the underlying structure of variables. For this study, promax rotation was used in conjunction with the maximum likelihood method because correlations were estimated between factors from the concept of SIT. The number of factors was determined based on Kaiser’s criterion (eigenvalues > 1) as well as the results of parallel analyses of the eigenvalues and squared multiple correlations (SMC) in the diagonal of the correlation matrix [30, 31].

To rate relevance per each factor for three groups of stakeholders, we checked Pearson’s chi-square and Cramer’s V as effect sizes for chi-square. We used a benchmark to interpret effect sizes for chi-square of around 0.10, 0.30 and 0.50 as indicative of negligible practical importance, moderate practical importance and crucial practical importance [32, 33].

Then we performed confirmatory factor analysis (CFA) to determine whether the data confirmed the model from the EFA. We asked the different cohort (the fourth year health professions students (*n* = 259) of the same university in December 2018) to answer the questionnaire. Then we used the following fit indices and criteria: chi-square divided by the degrees of freedom (CMIN/df) is < 2; the goodness-of-fit index (GFI) is > 0.90; the comparative fit index (CFI) is > 0.90; the root mean square residual (RMSEA) is < 0.1; and the PCLOSE value is > 0.05 [34].

In addition, we calculated correlations between the factor scores and the overall judgment and Cronbach alphas to indicate the internal consistency of the scale. Finally, we performed structural equation modeling to test a model incorporating all the factors and the decisions, with fit indices and criteria that were consistent with those listed above for the CFA.

We used SPSS 25.0 (IBM) and HAD 16.10 for calculations in the development phases and EFA, and Amos 11.0 (IBM) for CFA.

### Ethical approval

This study was approved by the Institutional Review Board of Shinshu University (#3719). Data was accessible only to the researchers.

## Results

### Instrument development

Of 30 panelists, 28 (93.3%) returned a fully completed questionnaire throughout the process. In the first Delphi round, the 27 items with the accepted ratings were sustained. Five items were reworded based on panelists’ suggestions. Of three new items proposed by panelists, we included two in the list. Another item was not proposed to include because it was considered to be similar in meaning to another one.

In the second Delphi round, all items had standard deviations < 1.25. As panelists proposed no additional item and provided no other negative responses, we concluded that no more round was necessary and consensus was reached. Therefore, we obtained a 16-item instrument for evaluating students’ perceptions regarding their own social interdependence attitude in collaborative learning.

### Construct validity and reliability

We received completed questionnaires from 239 fourth year health professions students (98 males and 141 females). The detailed breakdown of their specialties were medicine (*n* = 103), nursing (*n* = 65), occupational therapy (*n* = 17), physical therapy (*n* = 20), and medical technician (*n* = 34). Total response rate was 90.5%.

In the EFA, the Kaiser–Meyer–Olkin (KMO) measure of sampling adequacy was 0.913, which was satisfactory. Bartlett’s test of sphericity was significant with *p* < 0.001 (χ^2^ = 1838.56, df = 120). As per the determination of the numbers of factors, Kaiser method and parallel analysis of SMC suggested the three-factor structure, while parallel analysis of eigenvalues suggested the two-factor structure (Table 1). Since the concept of social interdependence theory comprised of three components (boundary, means, outcome), we tentatively adopted the three-factor model with factor loadings of ≥0.4 and the final decision of the structure would be decided based on the results of CFA. These factors were labeled as follows: boundary interdependence, means interdependence, and outcome interdependence. With regard to power calculations, all factors showed no significant differences between the three stakeholder groups. Effect sizes of chi-square were moderate to high [33]. The results are shown in Table 2.

**Table 1:** Determination of the number of factors

Number of factors (up to 5)	Eigenvalues	Cumulative contribution ratio (%)	Parallel analysis (Eigenvalues)	SMC diagonal	Parallel analysis (SMC diagonal)
1	6.787	45.248	1.493	6.293	0.597
2	1.419	54.707	**1.374**	0.919	0.452
3	**1.112**	62.118	1.282	0.637	**0.350**
4	0.732	67.000	1.217	0.230	0.285
5	0.704	71.692	1.145	0.144	0.223

**Table 2:** Pearson chi-square and effect sizes (ratings of relevance per factors for three groups of stakeholders)

	x^2^	df	effect size
boundary	26.04	28	0.682
means	14.083	14	0.501
outcome	10.798	10	0.439

With regard to the CFA, we received completed questionnaires from 228 students (response rate 88%). It was expected to demonstrate a suboptimal fit of conceptual framework of social interdependence as described in the Method section. Thus we removed one item (“My peers rely on me for materials, methods, and other things that they need”) from the instrument because it showed a high correlation (r > 0.7) with another item and the modification indices indicated the item that could be removed to achieve a better fit of the model. Subsequently, we generated alternative models which were consistent with social interdependence theory, and evaluated those models with stepwise testing. The final result demonstrated that a three-factor model with 15 items provided an excellent fit with CMIN/df = 1.838, GFI = 0.924, CFI = 0.951, RMSEA = 0.061, and PCLOSE = 0.121. EFA of this model was also satisfactory as KMO measure was 0.877 and Barrett’s test was significant with *p* < 0.001 (χ^2^ = 1479.583, df = 105). The final version of the instrument, the SOcial interdependence in Collaborative learning Scale (SOCS), which was comprised of 15 items, is as shown in Table 3 with their factor loadings of the pattern matrix.

**Table 3:** The final version of the instrument

	Items	Factor 1 (boundary)	Factor 2 (outcome)	Factor 3 (means)
1	I hope my learning group is superior to others.	**0.768**	−0.106	0.066
2	When there are different opinions, I would like to coordinate them.	**0.742**	−0.274	0.239
3	For me, it is important to maintain harmony within the group.	**0.647**	0.187	−0.119
4	I incorporate the advice of others when preparing a study plan.	**0.625**	0.127	−0.012
5	Group members should carefully summarize each other’s arguments.	**0.615**	−0.034	0.033
6	Discussions with other members who have different opinions will improve me.	**0.589**	0.267	−0.061
7	I try to share my own thoughts and materials if they are useful to other students.	**0.585**	0.157	0.023
8	I have respect for the others with whom I interact.	**0.551**	0.241	−0.038
9	It is a good idea to share the tasks for more efficient group work.	**0.442**	0.214	−0.02
10	I can learn important things from other students.	−0.108	**0.824**	0.116
11	It is a good idea for students to help one another in their studies.	0.076	**0.768**	−0.015
12	We learn numerous important things from one another.	0.066	**0.763**	0.047
13	My peers rely on my information and advice.	−0.026	0.019	**0.831**
14	My peers rely on my presence as well as my help and support.	0.004	0.08	**0.715**
15	I draw conclusions from information in group discussions.	0.094	0.091	**0.553**

We also tested other models based on the correlation data and theoretical assumptions. Since we confirmed that the three-factor model yielded a better fit (Table 4), we adopted the three-factor model above. The correlations between the factors were moderate, varying 0.358 to 0.672 (Table 5). Alpha-coefficients for all factors indicated high internal consistency of all the factors (outcome = 0.818, means = 0.866, boundary = 0.811).

**Table 4:** Results on confirmatory factor analysis with one to four- factor solutions (*n* = 228)

Number of factors	x2	df	*p*	CMIN/df	GFI	CFI	RMSEA	PCLOSE
1	514.2	90	0	5.713	0.725	0.7	0.144	< 0.001
2	265.6	89	0	2.985	0.857	0.875	0.094	< 0.001
**3**	**150.7**	**82**	**0**	**1.838**	**0.924**	**0.951**	**0.061**	**0.121**
4	160.7	83	0	1.936	0.917	0.945	0.064	0.059

**Table 5:** Correlations between factors (*n* = 228)

	outcome	means	boundary
outcome	1	0.410	0.358
means		1	0.672
boundary			1

## Discussion

This paper describes the development and validation of an instrument to measure social interdependence attitude of students in collaborative learning approaches. We used the modified Delphi method to establish a draft questionnaire by gathering different types of stakeholders in health professions education as panelists. The questionnaire was subsequently tested for construct validity. The CFA yielded a three-factor model with an excellent fit and high reliability of each factor.

The resulting instrument appears to reflect the characteristics of collaborative learning. In PBL, for example, as students work together in PBL tutorials, they may develop interdependent relationships facilitating learning and motivation [35]. Positive interdependence in PBL is fostered by creating instructional materials and more importantly learning materials [8]. The learning materials distributed throughout the group are interrelated in material interdependence. Thus, each student is expected to contribute actively to solve the whole problem; the problem cannot be solved when it is done by one student alone without collaborating with the others. In other words, we confirmed construct validity as well as internal consistency of the social interdependence in collaborative learning scale (SOCS). No other instrument to measure social interdependence exists in the literature.

It was characteristic that new items regarding respondents’ improvement were suggested (e.g. Discussions with other members who have different opinions will improve me) by stakeholders, while items regarding strong belonging to a team were deleted (e.g. If a team member fails their examination, I feel responsible for it.) during the Delphi procedures. This change represents the characteristics of social interdependence in collaborative learning in comparison to other fields. In many educational setting of higher education, summative assessment of each student is inevitable. Although some collaborative learning approaches (such as TBL) combine inter-group assessment and individual assessment, they will be finally graded individually. Thus they are forced to their own improvement in some way or other. In order to engage learners in improvement of team work rather than personal improvement, implementation of assessment processes per learning groups as well as the elaboration that learning tasks to collaborate with each other have to be related to the outcome.

With regard to the correlation between factors, three factors were correlated each other. Especially, outcome and boundary were correlated higher. The relatively close and mutual association of each factor is consistent with previous research on social interdependence theory. Stronger positive outcome interdependence, such as mutual learning goals, is associated with greater perceived entitativity of a group [36]. It is also associated with stronger social identity derived from group membership and self-esteem of each member (i.e. enhancement of boundaries [37]). Furthermore, task creates outcome through enhancement of responsibility [38] while dissimilarities of tasks lead to perceived boundaries [39]. These findings mean close relations between three subcategories, and our model shows good validity to assess these subcategories by comprehending such interactions.

### Limitations

There are several limitations to this study. Firstly, there would be room for increasing the number of panelists. For Delphi studies, there is no uniform rule regarding the number of panelists [40]. While more than 20 panelists have been recommended [28], too many panelists would be at risk of drop-out. Also, while stakeholders were gathered internationally, respondent students in the validation phase were at one institute in Japan. Although it is unknown how culture affects social interdependence in collaborative learning, there is some evidence on the relations between culture and interdependence. For example, Hashimoto and Yamagishi [41] differentiated social interdependence perception into rejection avoidance and harmony seeking attitudes, and revealed that there was no difference in harmony seeking between Japan and the United States, while Japanese respondents showed higher rejection avoidance. In this sense, collaborative learning approaches that could arouse attitudes to avoid being a target of envy (e.g. the best contributor in a group is awarded personally) might cause a difference in scores of SOCS.

### Implications for future research

The current study developed an instrument for measuring social interdependence in collaborative learning, in order to enable to explore dynamics of social interdependence in these educational approaches. In the next steps, we should try to identify in which steps of specific collaborative learning approaches (e.g. PBL, TBL) positive interdependence is promoted or hindered, or to what extent social interdependence is associated with educational achievement. Future studies should be tackled with such questions with this instrument. Such verification studies in the different contexts may provide further validity evidence on this instrument.

Also, enhancing social interdependence in interprofessional education (IPE) will be of interest, because entering into interdependent relationships is one of important competencies in interprofessional work [7]. When schools and educators want to measure the efficacy of IPE, there is already a questionnaire for assessing readiness of IPE (RIPLS) [42]. However, its relation to social interdependence, especially about factors between them are still unclear. This instrument will be a help for future research and educational activities regarding interprofessional and related areas.

## Conclusion

In conclusion, the current study developed SOCS, a scale for measuring social interdependence in collaborative learning, by gathering opinions of international stakeholders. It also revealed construct validity and reliability. In addition, the results of the study seemed to present a characteristic of the direction of interdependence in collaborative learning that three categories affect each other. By using this instrument, teachers in health professions education can obtain additional findings for promoting collaborative learning environment.

## Data Availability

The datasets used and/or analyzed during the current study are available from the corresponding author on reasonable request.

## Abbreviations

CFA: Confirmatory factor analysis
CFI: Comparative fit index
CMIN/df: Chi-square divided by the degrees of freedom
EFA: Exploratory factor analysis
GFI: Goodness-of-fit index
IPE: Interprofessional education
KMO: Kaiser–Meyer–Olkin
PBL: Problem-based learning
RMSEA: Root mean square residual
SIT: Social interdependence theory
SMC: Squared multiple correlations
TBL: Team-based learning
